# The effectiveness of nature-based therapy for community psychological distress and well-being during COVID-19: a multi-site trial

**DOI:** 10.1038/s41598-023-49702-0

**Published:** 2023-12-16

**Authors:** Yeji Yang, Hyunjin Kim, Minjung Kang, Hyunjin Baik, Yunseok Choi, Eu-Jean Jang, Eun-Jin Chang, Sukyoung Yun, Miok Park, Eunyeong Park, Hojun Yun, Taek-Joo Lee, Yeong-Han Kwon, Kwang-Pyo Hong, Ai-Ran Lee, Songhie Jung, Tai-Hyeon Ahn, Hye-Young Jin, Kee-Hong Choi

**Affiliations:** 1 School of Psychology, Korea University, 145 Anam-ro, Seongbuk-gu, Seoul, 02841 Korea; 2 KU Mind Health Institute, Korea University, 145 Anam-ro, Seongbuk-gu, Seoul, 02841 Korea; 3 Korea Research and Institute for People and Environment, 246, Munjeong-ro, Songpa-gu, Seoul, 05737 Korea; 4 GRAMDESIGN, 225, Jangmal-ro, Bucheon, 14609 Korea; 5 Institute of Science and Natural Resources, Korea University, 145 Anam-ro, Seongbuk-gu, Seoul, 02841 Korea; 6 Department of Counseling Psychology, Korea Baptist Theological University, 190, Bugyuseong-daero, Yuseong-gu, Daejeon, 34098 Korea; 7 Department of Smart Green Care, Daegu Catholic University, 13-13, Hayang-ro, Hayang-eup, Gyeongsan, 38430 Korea; 8 Department of Smart Green City Industry Convergence, Korea Nazarene University, 48, Wolbong-ro, Seobuk-gu, Cheonan, 31172 Korea; 9 Department of Environmental Landscape Architecture, Joongbu University, 201 Daehak-ro, Chubu-myeon, Geumsan-gun, 32713 Korea; 10 Landscape Yeoleum, 65 Poeun-ro 2ga-gil, Mapo-gu, Seoul, 04026 Korea; 11 Hantaek Botanical Garden, 2, Hantaek-ro, Baegam-myeon, Cheoin-gu, Yongin, 17183 Korea; 12 Department of Horticultural Design, Shingu College, 377 Gwangmyeong-ro, Seongnam, 13174 Korea; 13 Korea Institute of Garden Design, 45, World Cup buk-ro 9-gil, Mapo-gu, Seoul, 03998 Korea; 14 Landscape Urban Planning, Department of Human Environment Design, Cheongju University, 298, Daeseong-ro, Cheongwon-gu, Cheongju, 28503 Korea; 15 Gardens and Education Research Division, Korea National Arboretum, 415, Gwangneungsumogwon-ro, Soheul-eup, Pocheon, 11186 Korea

**Keywords:** Psychology, Environmental social sciences, Health care

## Abstract

During the COVID-19 pandemic, the world population faced various mental health challenges, highlighting a need for new community-based psychosocial interventions. This study aimed to investigate the effectiveness and feasibility of Nature-Based Therapy (NBT) for the community experiencing psychological distress during the pandemic. A multi-site trial comparing NBT and control groups was conducted in Korea with 291 participants exhibiting mild to severe depression or anxiety. A total of 192 participated in 30 sessions of therapeutic gardening, while 99 remained in the control group. Psychological distress and well-being were assessed using seven measures of depression, anxiety, daily activity, life satisfaction, mindfulness, stress, and loneliness. The effect sizes (Cohen’s d) of NBT compared to the control group were medium to large: depression (0.583), anxiety (0.728), daily activity (1.002), life satisfaction (0.786), mindfulness (0.645), stress (0.903), and loneliness (0.695). Multilevel analysis revealed significant Time × Group interaction effects for all measures. Pearson correlation (r = − 0.28 to 0.71) showed that changes in all variables correlated significantly with each other, with small to large effect sizes. Therapeutic alliance at post-test positively moderated the intervention effects on the outcomes. We concluded that NBT is a promising psychosocial intervention for treating psychological distress for community dwellers.

## Introduction

Due to the COVID-19 pandemic, the global population has suffered from various mental health problems, and long-term consequences are prevalent. During the pandemic, more than half of the general population was affected by the COVID-19 outbreak at a moderate-to-severe level, and the global prevalence of depression, anxiety, and stress in the general population was 28%, 35%, and 53%, respectively^1,2^. Compared to pre-COVID-19, depression and anxiety symptoms were almost threefold higher during the pandemic^3,4^. In particular, those with pre-existing mental disorders have reported a greater psychological burden due to COVID-19 and poorer access to services and support^5–8^. Mental health professionals have warned about the long-term impact of COVID-19 on mental health and suggested the need for new community-based treatments for the public^6,9^. In addition, during the pandemic, physical activity also decreased due to social restriction, and as a result, the long-term physical and mental sequelae of COVID-19 became a serious public health problem^10,11^. To address the public mental health problem and long-term sequelae of the pandemic, sustainable psychosocial intervention is needed in the community level.

Nature-based therapy (NBT), also known as nature-assisted therapy, natural therapy, or green care, is an intervention based on experiences and activities in a natural setting specifically designed to improve human mental and physical health^12^. NBT includes horticultural therapy, therapeutic gardening, and natural environment therapy such as wilderness therapy, outdoor adventure therapy, forest bathing, etc.^13,14^. The positive effects of NBT on general human health and well-being have been supported by previous studies^15–18^. In particular, several studies highlight the effectiveness of nature-based activities and green spaces in reducing psychological distress and enhancing well-being through mental restoration^19,20^.

The definition of psychological distress varies across research fields, but in general, psychological distress refers to maladaptive psychological functioning in response to stressful and challenging life events^21^, or it is described as a state of emotional distress with symptoms of depression and anxiety^22^. Among psychological distress variables, numerous studies have consistently reported the positive effects of nature or NBT in reducing depression and anxiety^23–25^. Beyer et al.^26^ found that greater availability of green spaces in residential areas was associated with lower levels of depression and anxiety, and this relationship remained consistent after controlling for confounding factors. In the field of social sciences, well-being primarily refers to subjective well-being, which can be defined as individual's well-being based on their personal evaluation of their lives^27^. This is a multidimensional concept, encompassing not only the absence of negative factors but also positive reactions such as positive emotions and life satisfaction^28^. Regarding the association between well-being and NBT, studies have shown that increase time in green spaces is associated with improved well-being, life satisfaction and vitality (e.g., perceived level of energy and fatigue)^29–31^. Similarly, a study conducted with elderly residents of nursing homes found that an 8-week horticultural therapy led to significant improvement in their daily activity levels, which can be considered similar to vitality^32^. Additionally, NBT is associated with better social relationships^33,34^. Given that people with worse mental health seem to get more benefits from the positive effects of the green environment^20^ and that green spaces have been shown to buffer the adverse impact of social and economic inequality^35^, NBT can be an effective and efficient intervention for people experiencing psychological distress.

Studies conducted in South Korea consistently highlight the positive impact of NBT on psychological distress and well-being. The results of a domestic meta-analysis on horticultural therapy demonstrated moderate to large effect sizes in the social, emotional, and physical domains^36^. Specifically, a 12-session horticultural therapy for middle-aged Korean women resulted in reduced depression and anxiety levels, concurrently bolstering their self-identity^37^. Another 10-week NBT program for elderly Koreans with mental health problems lowered stress levels, indicated by decreased cortisol levels^38^. Moreover, participants in an 8-week CBT (Cognitive behavioral therapy)-based intervention program administered within a forest environment reported substantial enhancements in their quality of life compared to the control group^39^. These findings illustrate that NBT reliably enhances psychological well-being within the cultural context of South Korea.

Although NBT is regarded as an effective psychosocial therapeutic intervention, reviews and meta-analyses have pointed out the low quality of existing studies in terms of experimental design or analytical methods^16,40,41^, including lack of a control group, unclear intervention protocol, non-validated measures, small sample size, and absence of follow-up assessment^42–44^. In addition to the methodological limitations of existing NBT studies, the causal relationship between gardening and improved health outcomes should be cautiously interpreted because its mechanisms and pathways remain unclear^43^. Stress Reduction Theory^45^ is one theory that represents the mechanism of nature-based therapy. It proposes that nature has a calming effect which alleviate stress. Meta-analysis findings reveal that exposure to nature has stress-relieving effects, indicated by decreased cortisol levels, self-reported stress, blood pressure and heart rate variability^46^. NBT also incorporates mindfulness, which aids individuals to achieve mental clarity and relaxation^47^. Participants in mindfulness gardening programs reported increased ability to focus on themselves, find relief from daily challenges, and engage fully in the present moment with nature^48^. This aligns with Kaplan’s Attention Restoration Theory^49^, which suggests that nature facilitates effortless attention redirection, leading to the restoration of cognitive fatigue.

Psychological distress and well-being variables also seem to impact each other; in a study of walking therapy in nature for depression, positive mental health at the end of the intervention mediated the decrease in depressive symptoms^50^. Studies have highlighted the effects of physical activity on health. One study proposed that green areas could be a valuable resource for improving health by encouraging people to participate in physical activity more often^51^. Physiologically, the enhanced immune function has emerged as a promising mechanism for the central pathway between nature and health^52^. In addition to internal mechanisms, social cohesion is another factor that explains the benefits of nature-based group therapy by enhancing social relationships and reducing loneliness^40^. As such, there have been attempts to analyze the mechanism of NBT, but not many studies have been reported the results of empirical analysis of the relationship between changes in variables. In studies investigating psychological treatments often combined with nature, such as mindfulness-based therapy, a negative relationship has been consistently reported between negative emotions like depression, anxiety, and stress, and positive mental health variables such as life satisfaction and mindfulness^53–55^. For NBT to work effectively as a psychosocial intervention, it is necessary to examine whether the mechanisms and associations between the variables found in psychotherapy also can be applied to NBT.

In psychosocial intervention, the therapeutic alliance is considered an important factor for successful treatment. A therapeutic alliance generally refers to the positive and collaborative relationship between a therapist and client (or patient). Therapeutic alliances are regarded as an important aspect of both individual and group psychological treatments^56,57^. However, there is little known about therapeutic alliances in the context of NBT. In studies on wilderness therapy, the relationship between therapeutic alliances and outcomes has been controversial. One study suggested that therapeutic relationships were key change agents for participants^58^, but other studies reported non-significant effects of therapeutic alliances on treatment outcomes^59,60^. The Buddhist psychotherapy perspective emphasizes the therapeutic alliance in NBT, considering as a triangular relationship (therapist, client, nature), therefore, the client perceives and interacts with the natural environment as a real presence, not a psychological phenomenon^61^. Despite the importance of therapeutic alliances in NBT, the empirical evidence is insufficient. Nonetheless, the therapists’ ability to guide clients to engage in nature-based activities and build therapeutic alliances is considered important for better outcomes in NBT^62,63^.

Therefore, the present study has three objectives. First, we investigated the effects of nature-based therapy on the psychological distress and well-being of general public during the COVID-19 pandemic using a moderate sample and validated measures. We hypothesized that the psychological distress and well-being of participants in the therapeutic gardening program would significantly improve compared with those of the control group. Depression, anxiety, stress, and loneliness, which were prevalent negative emotional responses during the Covid-19 period^64^, were selected as variables for measuring psychological distress. Additionally, drawing on previous research we incorporated well-being variables encompassing vitality and life satisfaction. Moreover, considering that mindfulness components are often integrated into nature and are closely related to depression and anxiety, we included mindfulness as well-being measures. Second, we investigated the association between changes (post-score minus pre-score) in psychological distress and well-being. We hypothesized that changes in each psychological distress and well-being variable would associate significantly with each other. Finally, we examined the impact of therapeutic alliances on NBT. We hypothesized that the greater the level of treatment alliance, the greater the improvement in psychological distress and well-being. By examining these hypotheses, this study aimed to investigate how effective and feasible it is to provide NBT in the community during the COVID-19 pandemic for the people experiencing mental health challenges.

## Results

### Sample characteristics

Participants’ characteristics, including basic demographics, employment, marital status, mental disorder diagnosis, and education level, are presented in Table 1. Most participants were female (n = 225, 77.3%) and nearly half were married (n = 128, 44.0%). The mean age was 53.48 (SD = 24.05), with no statistical difference between the gardening (M = 52.21, SD = 24.04) and control (M = 56.04, SD = 23.99) groups. Among a total of the 291 participants, 192 were assigned to the gardening group and 99 to the control group. The Wilcoxon test was used to test the significance age difference between the two groups, since it did not satisfy the assumption of normality, and chi-square tests were conducted for other demographic features. The two groups did not statistically differ in gender, age, education, and mental disorder diagnosis, except for marital status [χ2 (4) = 9.973, *p* = 0.041]. There were no significant differences in the baseline mean scores for any of the psychological distress and well-being variables as a result of the independent t-tests (all *p* > 0.05).

**Table 1:** Baseline characteristics of participants.

Baseline Characteristics	Gardening Group (n = 192)	Control Group (n = 99)	t or z or χ2	*p*-value
Gender, n (%)			1.041	0.308
Men	47 (24.5%)	19 (19.2%)		
Women	145 (75.5%)	80 (80.8%)		
Age, M (SD)	52.21 (24.04)	56.04 (23.99)	− 1.55	0.12
Employment, n (%)			9.985	0.076
Unemployed	49 (25.5%)	24 (24.2%)		
Student	47 (24.55)	15 (15.2%)		
Homemaker	32 (16.7%)	16 (16.2%)		
Employed	33 (17.2%)	19 (19.2%)		
Other	14 (7.3%)	5 (5.1%)		
Unknown	17 (8.9%)	20 (20.2%)		
Marital Status, n (%)			9.973	0.041*
Never married	58 (30.2%)	19 (19.2%)		
Married	73 (38.0%)	55 (55.6%)		
Divorced	8 (4.2%)	1 (1.0%)		
Widowed	32 (16.7%)	14 (14.1%)		
Unknown	21 (10.9%)	10 (10.1%)		
Education, n (%)			9.062	0.170
No Education	10 (5.2%)	11 (11.1%)		
Elementary School (≤ 6 years)	32 (16.7%)	13 (13.1%)		
Middle School (≤ 9 years)	36 (18.8%)	11 (11.1%)		
High school (≤ 12 years)	50 (26.0%)	28 (28.3%)		
University/College Bachelor’s degree (≤ 16 years)	39 (20.3%)	20 (20.2%)		
Higher Education (> 16 years)	3 (1.6%)	5 (5.1%)		
Unknown	22 (11.5%)	11 (11.1%)		
Mental Disorder Diagnosis, n (%)			3.735	0.053
None	144 (75.0%)	84 (84.8%)		
Yes	48 (25.0%)	15 (15.2%)		
Psychological distress & Well-being measures, M (SD)				
MHS:D	17.3 (9.49)	18.0 (10.7)	− 0.541	0.59
MHS:A	16.9 (8.74)	17.8 (10.1)	− 0.812	0.42
CORE	15.0 (3.68)	14.4 (4.14)	1.23	0.22
SWLS	18.2 (6.43)	17.9 (7.02)	0.314	0.75
MAAS	56.6 (13.2)	55.6 (14.0)	0.573	0.57
PSS	20.5 (5.30)	21.1 (5.52)	− 0.78	0.44
ULS-8	11.1 (4.56)	10.7 (4.31)	0.697	0.49

t = t-value, χ2 = chi-square value, z = z value from two-samples Wilcoxon test. *p-value < 0.05. *M* and *SD* are used to represent mean and standard deviation, respectively.

*MHS:D* mental health screening tool for depressive disorders, *MHS* a mental health screening tool for anxiety disorders, *CORE* core life activities index, *SWLS* satisfaction with life scale, *MAAS* mindful attention awareness scale, *PSS* perceived stress scale, *ULS-8* a short form of the UCLA loneliness scale.

### Effects of gardening on psychological distress and well-being

**Figure 1:**
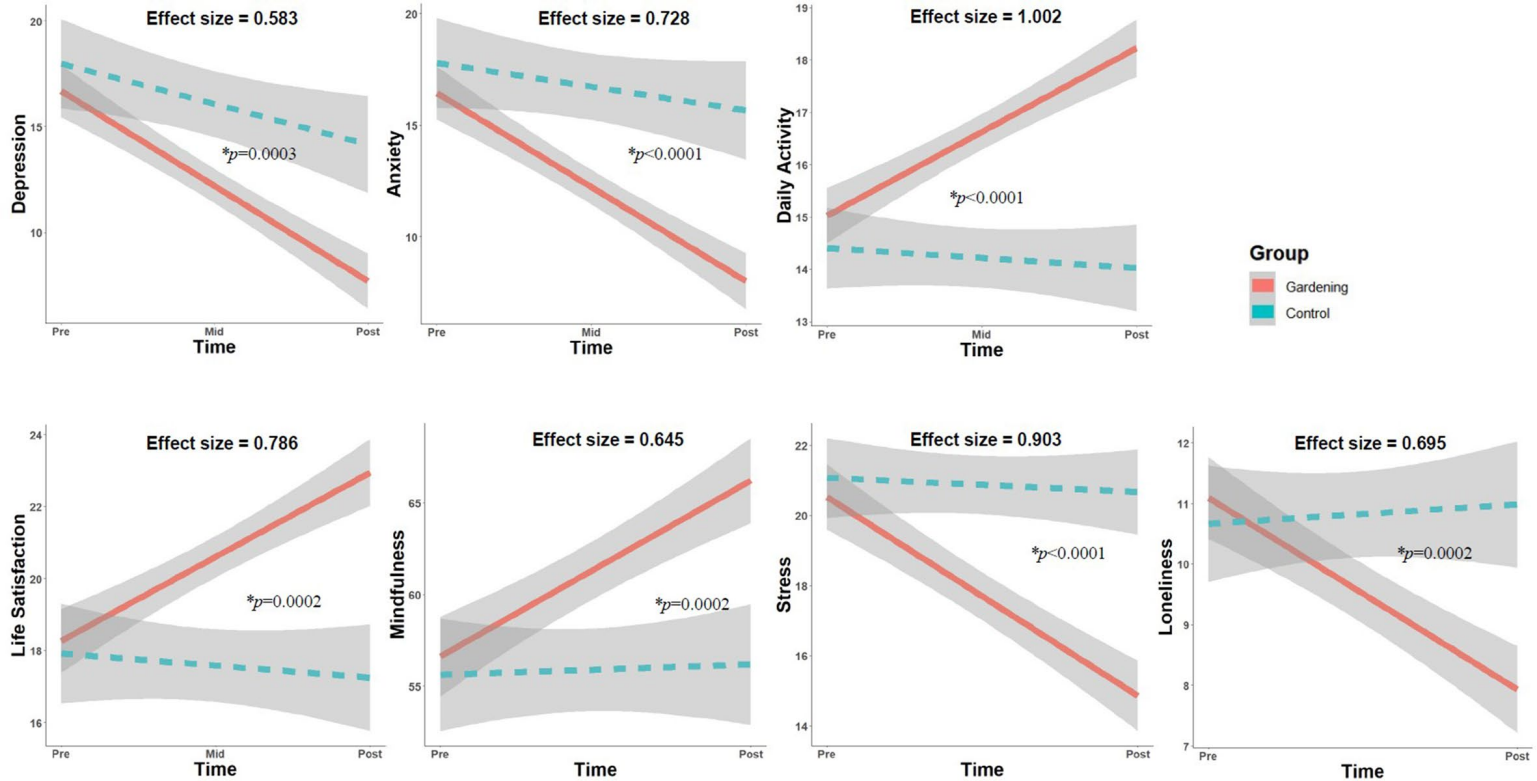
Significant interaction effects of study condition (Gardening vs Control) on mental health and well-being variables. Shaded areas represent the 95% confidence intervals. Effect sizes mean Cohen’s d value. *P*-values from ANCOVAs entering Marital Status as a covariate.

The results of the multilevel analysis are presented in Supplementary Table S1, and the mean scores at each time point, effect sizes (Cohen’s d), and statistical significance (*p*-value) of the ANCOVA are presented in Supplementary Table S2. Significant interaction effects were observed in the time × group (gardening vs. control) for all psychological distress and well-being measures (MHS:D, MHS:A, CORE, SWLS, MAAS, PSS, ULS-8) (all *p* < 0.01; Table S1). Specifically, the two groups did not statistically differ at baseline; however, the gardening group showed significant improvements in all psychological distress and well-being variables after the intervention, whereas the control group did not (Fig. 1). Effect sizes (Cohen’s d) were medium for the MHS:D (d = 0.583), MHS:A (d = 0.728), SWLS (d = 0.786), MAAS (d = 0.645), and ULS-8 (d = 0.695), and large for the CORE (d = 1.002) and PSS (d = 0.903). Even after controlling for the effects of marital status as a covariate, gardening effects were maintained for all psychological distress and well-being variables (Table S2). Although there were no significant differences between the groups in other demographic features, ANCOVA was further conducted after adjusting for age, sex, employment, education, and mental disorder diagnosis, considering the possibility of other potential confounders. And the results showed that there were still significant differences between two groups after the intervention even after adjusting demographic variables (all *p* < 0.05).

### Relationships between psychological distress and well-being variables

All difference scores (post-test minus pre-test) of the psychological distress and well-being variables correlated significantly with each other, with moderate-to-large effect sizes (all *p* < 0.01, Table 2). Specifically, depression, anxiety, and stress showed large correlation coefficients with statistical significance: depression-anxiety (r = 0.70), depression-stress (r = 0.66), and anxiety-stress (r = 0.70). Daily activity, life satisfaction, and mindfulness also had large positive correlations: daily activity-life satisfaction (r = 0.71), daily activity-mindfulness (r = 0.55), and life satisfaction-mindfulness (r = 0.61). Loneliness was significantly correlated with anxiety (r = 0.50), daily activity (r = − 0.50), and life satisfaction (r = − 0.55), with a large effect size.

**Table 2:** Means, standard deviations, and correlations of therapeutic alliances and difference scores of psychological distress and well-being variables.

Variable	*M*	*SD*	1	2	3	4	5	6	7	8	9
1. T1	39.60	10.14									
2. T2	45.16	12.66	0.33** [0.18, 0.46]								
3. AVE	42.04	9.46	0.78** [0.72, 0.84]	0.86** [0.81, 0.90]							
4. Depression	− 8.37	11.01	− 0.12 [− 0.27, 0.04]	− 0.38** [− 0.50, − 0.24]	− 0.33** [− 0.46, − 0.19]						
5. Anxiety	− 8.42	11.10	− 0.08 [− 0.23, 0.07]	− 0.40** [− 0.52, − 0.26]	− 0.31** [− 0.44, − 0.17]	0.70** [0.62, 0.77]					
6. Daily activity	3.31	4.77	0.01 [− 0.14, 0.17]	0.40** [0.26, 0.53]	0.27** [0.13, 0.41]	− 0.28** [− 0.42, − 0.13]	− 0.44** [− 0.55, − 0.31]				
7. Life Satisfaction	4.73	7.75	0.06 [− 0.10, 0.21]	0.52** [0.40, 0.63]	0.37** [0.23, 0.49]	− 0.36** [− 0.49, − 0.22]	− 0.40** [− 0.52, − 0.27]	0.71** [0.62, 0.78]			
8. Mindfulness	9.55	15.70	− 0.03 [− 0.20, 0.14]	0.52** [0.39, 0.64]	0.33** [0.18, 0.47]	− 0.35** [− 0.49, − 0.20]	− 0.39** [− 0.52, − 0.23]	0.55** [0.42, 0.66]	0.61** [0.50, 0.71]		
9. Stress	− 5.43	7.44	− 0.20 [− 0.36, − 0.03]	− 0.51** [− 0.63, − 0.37]	− 0.43** [− 0.56, − 0.29]	0.66** [0.56, 0.75]	0.70** [0.60, 0.77]	− 0.51** [− 0.63, − 0.38]	− 0.48** [− 0.60, − 0.34]	− 0.51** [− 0.63, − 0.38]	
10. Loneliness	− 3.28	4.98	− 0.01 [− 0.18, 0.15]	− 0.36** [− 0.50, − 0.21]	− 0.25* [− 0.39, − 0.09]	0.37** [0.23, 0.51]	0.50** [0.37, 0.61]	− 0.50** [− 0.61, − 0.37]	− 0.55** [− 0.65, − 0.42]	− 0.46** [− 0.59, − 0.30]	0.51** [0.37, 0.63]

T1: Therapeutic alliance score at mid-intervention, T2: Therapeutic alliance score at post-intervention, AVE: average of T1 and T2. *M* and *SD* are used to represent mean and standard deviation of difference scores, respectively. Each mental health and well-being variable means difference score (post-test minus pre-test). *p-value < 0.05, **p-value < 0.01.

### Effects of therapeutic alliance on psychological distress and well-being variables

The mean scores of therapeutic alliances for the gardening group at each time point and the results of the Pearson’s correlation analysis between therapeutic alliance and psychological distress and well-being variables are presented in Table 2. The mean therapeutic alliance score at T2 (M = 45.16, SD = 12.66) was higher than that at T1 (M = 39.60, SD = 10.14). The Pearson correlation results showed significant associations between the post-treatment (T2) therapeutic alliance scores and the intraindividual pre-post score differences in all the psychological distress and well-being variables (all *p* < 0.01): depression (r = − 0.38), anxiety (r = − 0.40), daily activity (r = 0.40), life satisfaction (r = 0.52), mindfulness (r = 0.52), stress (r = − 0.51), and loneliness (r = − 0.36). Similar results were observed in the correlation analysis between the average of mid- and post-treatment (AVE) therapeutic alliance scores and psychological distress and well-being variables, while mid-treatment (T1) therapeutic alliance scores did not significantly correlate with any variables. Given that significant associations were found between post-treatment (T2) therapeutic alliance scores and pre-post difference scores for all psychological distress and well-being variables, further multilevel moderation analysis was conducted. Therapeutic alliances (T2) had significant moderating effects on all measures of psychological distress and well-being (all *p* < 0.01). Table 3 presents the results of the moderation analysis for all variables.

**Table 3:** The result of moderation analysis of therapeutic alliance (T2).

	Effects Estimate	95% CI		SE	df	t	*p*
		Lower	Upper				
MHS:D							
Intercept	14.2162	10.0763	18.3564	2.104	305	6.7568	< 0.0001
Time	2.67	− 0.4676	5.8075	1.5945	305	1.6745	0.0951
Alliance	− 0.0594	− 0.1406	0.0218	0.0411	148	− 1.4453	0.1505
Time * Alliance	− 0.1455	− 0.2103	− 0.0807	0.0329	305	− 4.4185	< 0.0001*
MHS:A							
Intercept	14.972	10.7872	19.1568	2.1268	309	7.0397	< 0.0001
Time	3.1871	0.1324	6.2418	1.5525	309	2.0529	0.0409
Alliance	− 0.0726	− 0.1539	0.0086	0.0411	148	− 1.7664	0.0794
Time * Alliance	− 0.1567	− 0.2202	− 0.0932	0.0323	309	− 4.8556	< 0.0001*
CORE							
Intercept	13.1144	11.4013	14.8275	0.8707	313	15.0622	< 0.0001
Time	− 1.2887	− 2.7302	0.1528	0.7326	313	− 1.7591	0.0795
Alliance	0.0801	0.0447	0.1155	0.0179	148	4.4677	< 0.0001
Time * Alliance	0.0634	0.0344	0.0924	0.0148	313	4.2969	< 0.0001*
SWLS							
Intercept	14.5842	11.6622	17.5062	1.4851	313	9.8204	< 0.0001
Time	− 4.0452	− 6.2787	− 1.8117	1.1352	313	− 3.5636	0.0004
Alliance	0.1398	0.0777	0.2019	0.0314	148	4.4504	< 0.0001
Time * Alliance	0.1391	0.0943	0.1839	0.0228	313	6.1087	< 0.0001*
MAAS							
Intercept	52.991	45.4336	60.5483	3.82	130	13.8721	< 0.0001
Time	− 19.009	− 28.2457	− 9.7713	4.6691	130	− 4.0711	0.0001
Alliance	0.216	0.0670	0.3650	0.0753	122	2.8696	0.0048
Time * Alliance	0.619	0.4289	0.8084	0.0959	130	6.4503	< 0.0001*
PSS							
Intercept	22.4525	19.7021	25.2030	1.3902	129	16.151	< 0.0001
Time	6.7768	2.4720	11.0815	2.1757	129	3.1147	0.0023
Alliance	− 0.1104	− 0.1627	− 0.0580	0.0264	122	− 4.1754	0.0001
Time * Alliance	− 0.2505	− 0.3332	− 0.1678	0.0418	129	− 5.9906	< 0.0001*
ULS-8							
Intercept	13.4108	11.2434	15.5783	1.0962	139	12.2336	< 0.0001
Time	1.9193	− 1.0938	4.9324	1.5240	139	1.2594	0.21
Alliance	− 0.0897	− 0.1330	− 0.0465	0.0219	130	− 4.1068	0.0001
Time * Alliance	− 0.1081	− 0.1698	− 0.0464	0.0312	139	− 3.4637	0.0007*

Post-intervention. SE = Standard Error. df = degree of freedom. t = t-value. *p-value < 0.01.

*MHS:D* mental health screening tool for depressive disorders, *MHS:A* mental health screening tool for anxiety disorders, *CORE* core life activities index, *SWLS* satisfaction with life scale, *MAAS* mindful attention awareness scale, *PSS* perceived stress scale, *ULS-8* a short form of the UCLA loneliness scale.

## Discussion

This study examined the effects of nature-based therapy on the psychological distress and well-being of individuals with depressive and anxiety symptoms during the COVID-19 pandemic. Overall, NBT showed significant treatment effects on psychological distress and well-being compared with the control group. As hypothesized, all psychological distress and well-being variables of participants in the therapeutic gardening program significantly improved compared with the control group. This result is consistent with many previous studies reporting the positive effects of NBT on psychological distress and well-being^42–44,65^. The effects sizes found in this study, which are 0.583 for MHS:D, 0.728 for MHS:A, 1.002 for CORE, 0.786 for SWLS, 0.645 for MAAS, 0.695 for ULS-8, and 0.903 for PSS, were similar or larger than the effect sizes (0.35 to 0.95) reported in previous meta-analysis studies of NBT^16,41,43^.

Regarding psychological distress, the same result was derived from previous studies reporting that NBT showed a larger effect size for anxiety than for depression^16,42^. However, since many studies have consistently reported the effectiveness of NBT for depression, it would be appropriate to consider NBT as effective for general mood disorder symptoms rather than concluding that it is more effective for anxiety than for depression. Interestingly, stress was the most improved variable among negative affect variables (depression, anxiety, and loneliness). Considering that previous studies have continuously reported the effectiveness of NBT in reducing stress, stress seems to be easily alleviated by NBT. In addition to self-reported stress levels, several studies have investigated the physiological effects of NBT in reducing stress using EEG, blood pressure, pulse rate, and the immune system^66,67^. Moreover, NBT was found to be effective in improving the quality of life and reducing burnout in stress-related mental illnesses^65^. Although the mechanism of NBT in relieving stress should be further studied, from the Stress Reduction Theory’s view, its effects can be explained by the fact that restorative responses to non-threatening nature have benefited human beings during evolution, including the rapid attenuation of stress responses^45^.

Positive mental health and well-being aspects such as daily activity, life satisfaction, and mindfulness improved significantly, as did negative affect. In line with previous studies, NBT had a positive impact on life satisfaction. Interestingly, despite the absence of mindfulness-focused practices within the therapeutic gardening program, participants' levels of mindfulness improved. This could be explained by the possibility that similar mental process to mindfulness occur during nature-based activities, or it may be because the attention to the nature stimuli reduces mind wandering^68,69^. In addition to the several studies suggesting that NBT is beneficial for increasing vigor or physical activity, daily activity level (vitality) showed the greatest improvement in this study among all psychological distress and well-being variables. We used the Core Life Activities Index (CORE) for vitality, which assesses five aspects of daily activity: sleep, eating, physical activity, spending time with friends and family, and new learning, rather than physical activity or vigor. NBT can be considered more effective in enhancing vitality indicating the energy in daily life aspect, rather than to the vitality defined in terms of physical activity levels observed in other studies^70,71^. Loneliness, which represents social aspects of well-being, significantly decreased, showing results similar to those of other studies on the psychosocial effects of NBT^72,73^. In the case of group-based NBT, gardens serve as vital social arenas, thus appearing to offer additional psychosocial benefits, including reduced loneliness and improved social bonds, compared to home gardening or individual horticultural therapy^73,74^. In the study comparing the effects of individual and group horticultural interventions, group-based intervention showed significantly higher improvements in the socially related sub-scores of the quality of life and emotional intelligence (social score, interpersonal score, and empathy score)^74^. Alternatively, given the significant moderating effect of the therapeutic alliance on the reduction of loneliness, the social relationship or bonding with the therapist may have influenced the decrease in loneliness. Since our study aimed to examine the effects of the NBT as compared to the control condition, it would be beyond the scope of the current study to conclude whether the benefits of the NBT were associated with socializing components or exposure to nature. Considering the previous studies indicating that social interactions were significantly higher after participation in social and therapeutic horticulture or gardening, it should be investigated whether enhanced social interaction during the NBT serves as a therapeutic mechanism along with other potential contributing factors, such as mindfulness^68,69^, and exposure to nature in a future study.

We also investigated the association between changes (post-score minus pre-score) in seven psychological distress and well-being variables: depression, anxiety, daily activity, life satisfaction, mindfulness, stress, and loneliness. As hypothesized, significant correlations were found between all the variables. This implies that people experience changes in psychological distress and well-being through comprehensive interactions and not independently. Psychological distress variables (depression, anxiety, and stress) showed large correlations with each other, while positive mental health and well-being variables (daily activity, life satisfaction, and mindfulness) correlated with large effect sizes. This is probably because people suffering from psychological distress experience a combination of negative emotions and improvements occur simultaneously through the intervention. Similarly, there were significant associations among depression, anxiety, and stress in studies on the psychological impact of COVID-19^75,76^. Regarding positive mental health and well-being variables, it is probable that people experience vitality and mindfulness while participating in gardening, which improves their life satisfaction. In a study on the effectiveness of therapeutic gardening as behavioral activation, vitality mediated the improvement of quality of life, depression, and anxiety^77^. The relationship between psychological variables and the mediating effects of nature-based therapy should be further investigated. The current research team is currently conducting follow-up research with a specific focus on investigating the mechanisms involved.

Finally, in line with our hypothesis, therapeutic alliances positively affected psychological distress and well-being. Specifically, therapeutic alliance scores at T2 (post-test) were higher than at T1 (mid-test) and had a greater impact on the intervention. This finding is inconsistent with the literature suggesting that early alliance scores are predictive of treatment outcomes^78^. One potential explanation is that the improvement in psychological distress and well-being as the intervention progressed may have enhanced the therapeutic alliance. A therapeutic alliance predicts successive changes in symptoms, and prior symptom changes also affect the therapeutic alliance^79^. This suggests that the therapeutic alliances and treatment effects mutually affect each other. Although alliances in group therapy have a smaller effect than in individual therapy, owing to the dynamics of the group^56^, group cohesion is still an important factor in the outcome of nature-based group therapy^80^. Therefore, therapists must create supportive and connected group environments to achieve better outcomes.

This study had several limitations. First, regarding the experimental design, we did not adopt a randomized controlled trial, and the discrepancy in the number of participants between groups necessitates caution in interpreting the representativeness of the sample and the generalizability of the study results. Since the research was conducted during the pandemic period, it was difficult to recruit participants and randomize them. While COVID-19 had subsided, vulnerable populations, such as those with mental health problems or the older adults, still had a fear of face-to-face contact with people and hesitant to return to social life^81^. Since people were engaged in limited face-to-face activities at their institutions, there were significant concerns about randomizing them to nature-based interventions or treatment as usual. Thus, it was deemed unethical to randomize them. To cope with this limitation, we recruited participants (N) that could derive sufficient statistical power, and attempted to control for individual and group variables using a multilevel model. To provide robust evidence for NBT, studies should have larger sample sizes to avoid type II errors, accurately discover differences between groups, and use reliable measures to ensure strong internal validity^15^. Therefore, we tried to recruit as many participants (N) and employed validated measures. Also, non-randomized controlled designs could provide adequate evidence as alternatives to RCT, and is more convincing when confounders are well-understood, measured and controlled, there is evidence for causality between intervention and outcomes, and effect sizes are large^82^. In this regard, we selected outcome variables by investigating various existing literatures. We have tested differences in demographic data and psychological distress and well-being variables at baseline between the two groups and found no differences, except for marital status. All statistical analyses were conducted after controlling for marital status, and significant large effect sizes were obtained. Nevertheless, there still is a possibility that the motivation of some gardening group participants affected the treatment effect. Second, we were unable to conduct follow-up assessments because of limitations in the study duration. Conflicting results have been reported regarding the long-term effects of nature-based treatments^13,43^, and it is necessary to conduct a follow-up assessment in future studies. Finally, since the intervention was conducted at a time when social restrictions caused by the COVID-19 were gradually eased, there may have been more psychosocial effects. Therefore, it is needed to be cautious in interpreting the effectiveness of NBT during this period.

Despite these limitations, the results of this study contribute to the effectiveness of nature-based therapy, represented by a therapeutic gardening program, as a psychosocial intervention for community dwellers suffering from psychological distress during COVID-19. There are very few experimental studies in the field of NBT research with a sample size exceeding 200^14,16,43^, and during the COVID-19 pandemic, most studies investigating the impact of nature-based activities on mental health and well-being were predominantly survey-based^83,84^. In this regard, the current study resulted in the applicability and feasibility of NBT to local communities by conducting experimental research involving a moderate sample recruited from multi-sites. Additionally, from a methodological standpoint, we tried to control for confound variables, such as site and demographic characteristics, that could potentially influence on the treatment outcomes, with the aim of rigorously examining the effectiveness of NBT.

We concluded that nature-based therapy is an effective and feasible psychosocial intervention and applicable for improving community’s psychological distress and well-being. In addition to the applicability of NBT, the current study explored the significant interactive association between changes in psychological distress and well-being, which can provide implication for future mechanism studies. Moreover, a later therapeutic alliance was found to play an important role in the intervention outcomes of NBT, similar to other psychosocial treatments. Therefore, future studies on nature-based therapy should focus on the effect mechanism, research methodology of random assignment, therapist competency, and adherence to evidence-based treatments.

## Method

### Study design

We employed a multi-site experimental design with repeated measures to examine the effects of the therapeutic gardening program on the participants’ psychological distress and well-being. Depending on the recruitment center, participants were assigned to the gardening or control group. The gardening group participated in nature-based therapy for 30 sessions, whereas the control group did not receive any nature-based activities or intervention. The same outcome measures were employed for both groups except for therapeutic alliances with therapists.

### Participants

All experimental procedures and protocols of the current study were approved by the Korea University Institutional Review Board (protocol no. KUIRB-2022-0218-03, 05/04/2022), and all methods were performed in accordance with the relevant guidelines and regulations. A total of 291 participants were recruited from 11 institutions across Korea, including senior welfare centers (n = 96, 33%), medical centers (n = 67, 23%), local universities (n = 45, 15.5%), botanical gardens (n = 33, 11.3%), special schools (n = 17, 5.8%), community center (n = 19, 6.5%), and mental health center (n = 14, 4.8%). Since this study was multi-site trial, recruitment period (from May to June 2022) was slightly various depending on each institution’s situation. Even though recruitment period was different, recruitment process, screening tools, measures, and therapeutic gardening program were conducted identically for all the institutions. The inclusion criteria were as follows: (1) age over 13, (2) had mild depressive or anxiety symptoms. For participants under the age of 18, parental consent was obtained to ensure their involvement in the study. Screening for the depression and anxiety was based on the Mental Health Screening Tool for Depression (MHS:D)^85^ and the Mental Health Screening Tool for Anxiety (MHS:A)^86^. Participants who scored higher than 8 on the MHS:D or 10 on the MHS:A, indicating mild depression and anxiety levels, respectively, were determined to be eligible to participate in the current study. Participants were excluded if they had: (1) mobility problems, (2) communication difficulties, or (3) severe mental illness thereby hindering program participation. The assessment of the exclusion criteria was evaluated through interviews by experts such as social worker, horticultural therapist, and psychologist. Based on diagnostic interviews, individuals with mental disorders who demonstrated the ability to maintain their daily functioning as outpatient patients were included as participants for the study.

After determining eligibility, participants were assigned to either the therapeutic gardening group (n = 192) or the control group (n = 99) according to the situation at the recruitment center. An adequate sample size was estimated using G*power version 3.1. software^87^ based on an effect size of 0.642, which is the mean value of the five mental health and well-being-related effect sizes from our previous study^44^. In our previous study, we examined the feasibility and preliminary effects of therapeutic gardening through single-group multilevel analysis, and as a result, the effect sizes of mental health variables were small to medium (0.40 to 0.84)^44^. According to the power analysis, a sample size of 34 in each group would provide a power of 0.95 to yield statistically significant results, therefore the number of recruitments was sufficient to yield statistically significant results. All participants were enrolled after providing written informed consent. Each time the subjects completed a self-rated assessment, a compensation of KRW 10,000 was rewarded, and a mental health report was provided. The assessment was conducted under the management of gardening therapists trained by a clinical psychologist. The study was registered with the Clinical Research Information Service (CRIS) and conformed to the World Health Organization International Clinical Trials Registry Platform (WHO-ICTRP) (registration no. KCT0008085, 06/01/2023).

### Intervention

The gardening program was conducted from June to November 2022 as a group therapy with 30 sessions over 15 weeks, based on the suggested manual of the Korea National Arboretum in 11 green-area sites nationwide (e.g., botanical gardens, gardens in medical centers, and green areas in universities). The gardening program took place twice a week, with each session lasting around 2 h. The contents of the gardening program are presented in Supplementary Table S3. The gardening program consisted of 85% gardening activities (e.g., planting, fertilizing, repotting, mulching) and 15% events (e.g., enjoying herb tea, flower arrangement, and picnics in the garden). The content of the therapeutic gardening program sessions was reviewed and discussed with experts in clinical psychology and horticultural therapy in advance but was modified flexibly according to each site’s circumstances, such as participant characteristics (e.g., older adults) and weather problems.

In the control group, participants were not provided any nature-based activities or intervention. However, participants recruited from community centers participated in daily activities provided by their centers as usual (TAU) without engaging in any type of nature-based intervention. For example, older adults in senior center participated in group aerobic, singing or reading classes provided by community center as usual. In the case of students, they received vocational training such as computer programming, cook training, and service training. Participants who were recruited from medical center or mental health care center were provided medical care as usual.

### Measures

The participants completed seven self-rated psychological distress and well-being measures. Pre-test was conducted before the gardening program had begun (at baseline) and post-test was conducted after the final session (at the end of the program). In the gardening group, depression, anxiety, daily activities, and life satisfaction were assessed three times to examine the change trends (at baseline, in the middle of the program, and at the end of the program). Therapeutic alliances were assessed twice, at mid- and post-intervention, in the gardening group. Sociodemographic information such as gender, age, employment, marital status, education, and the presence of mental disorders was collected at baseline.

#### Mental health screening tool for depressive disorders (MHS:D)

Depressive symptoms were assessed using the Mental Health Screening Tool for Depressive Disorders (MHS:D)^85^, which was developed for the early screening of patients with major depressive disorder with relatively high accuracy in the primary medical setting. It is a 12-item questionnaire scored on a 5-point Likert scale. Higher scores indicated more experience with symptoms associated with the diagnostic features of major depressive disorder (MDD) over the past two weeks. Total MHS:D scores can be interpreted as the minimal range (0–8), mild (8–12), moderate (12–20), and severe (> 20) depressive symptoms. MHS:D was developed in two versions, online and offline, both of which demonstrated excellent internal consistency (Cronbach’s α, offline version:0.943, online version:0.945)^85^ and both were utilized in this study for the convenience of the participants.

#### Mental health screening tool for anxiety disorders (MHS:A)

Severities of anxiety symptoms were measured with Mental Health Screening Tool for Anxiety Disorders (MHS:A)^86^, developed for the timely screening of generalized anxiety disorder (GAD) with relatively high accuracy in the primary medical setting. MHS:A self-report measure that assesses how often symptoms related to generalized anxiety disorder have been experienced in the past two weeks. MHS:A has 11 items scored on a five-point Likert scale (0 = never, 4 = always). Higher total scores indicated a higher level of anxiety symptoms, interpreted as minimal range (0–10), mild (10–20), moderate (20–30), or severe (> 30) anxiety symptoms. MHS:A has been reported to have excellent internal consistency in both online and offline versions (Cronbach’s α, offline version: 0.957, online version: 0.956)^86^ and both versions were used in this study at the convenience of the participants.

#### Core life activities index (CORE)

The Core Life Activities Index (CORE), developed by the current research team, was administered to assess the level of vitality in daily activities over the past week. This measure includes five questions about engaging in everyday activities such as sleep, eating, and physical activity, scored on a five-point Likert scale (1 = never, 5 = always). Higher scores indicated greater engagement in daily activities, which can be interpreted as higher vitality. CORE has previously shown good internal consistency in a preliminary study on the psychological effects of COVID-19 in Korea (Cronbach’s α = 0.77)^75^.

#### Satisfaction with life scale (SWLS)

Subjective life satisfaction was assessed using the Korean version of the Satisfaction with Life Scale (K-SWLS)^88^, which was validated using the Korean version of the original Satisfaction with Life Scale (SWLS)^89^. The SWLS is a 5-item measure with a seven-point Likert scale (1 = strongly disagree to 7 = strongly agree). Higher scores indicated greater satisfaction with one’s personal life. K-SWLS has shown good internal consistency in the validation study (Cronbach’s α = 0.85–0.90)^88^.

#### Mindful attention awareness scale (MAAS)

Mindfulness was assessed using the Korean Version of Mindful Attention Awareness Scale (K-MAAS)^90^, which is a validated version of the original Mindful Attention Awareness Scale (MAAS)^91^. The MAAS is a 15-item measure that assesses mindful attention and awareness in one’s daily life using a 6-point Likert scale (1 to 6), with higher scores indicating greater mindfulness. The K-MAAS reported significant internal consistency in a validation study (Cronbach’s α = 0.87)^90^.

#### Perceived stress scale (PSS)

The level of perceived stress in daily life was assessed using the Korean Version of the Perceived Stress Scale^92^, which is a validated version of the original scale^93^. The PSS consists of 10 items that measure the degree to which individuals have perceived and interpreted subjective stress over the past month, using a five-item Likert scale (0 to 4). A higher total score indicated a greater degree of stress experienced by the individual. The Korean version of the PSS showed good internal consistency in a validation study (Cronbach’s α = 0.82)^92^.

#### A short form of the UCLA loneliness scale (ULS-8)

Loneliness was measured using a short form of the UCLA Loneliness Scale (ULS-8)^94^, the abbreviated version of the 20-item UCLA Loneliness Scale^95^, which is the most commonly used measure of loneliness. This 8-item measure assesses the subjective sense of being separated from others and is scored on a four-point Likert scale (0 to 3). A higher total score indicated a greater degree of loneliness. The ULS-8 reported good internal consistency in a validation study (Cronbach’s α = 0.84)^94^.

#### Working alliance inventory-short revised (WAI-SR-K)

The Korean version of the Working Alliance Inventory-Short Revised (WAI-SR-K)^96^ is the Korean version of the Working Alliance Scale-Short Revised version^97^. The WAI-SR is a 12-item scale that assesses therapeutic alliances. It consists of three subscales with four items respectively: agreement on the goals of therapy, agreement on the tasks of therapy, and the development of an affective bond between therapists and clients. A higher total score indicated a greater degree of therapeutic alliance with the therapist experienced by the client. The WAI-SR-K showed excellent internal consistency in the validation study (Cronbach’s α = 0.93)^96^.

### Statistical analysis

An independent team performed all statistical analyses. Independent t-tests and chi-square tests were conducted for continuous and categorical variables, respectively, to examine baseline differences in demographic characteristics and psychological distress and well-being variables between the two groups (gardening group vs. control group). Among the demographic features, marital status was found to be non-equivalent between the groups; therefore, ANCOVA was conducted to re-analyze the treatment effects, considering marital status as a covariate. If the data did not satisfy the assumptions such as normality and homogeneity of variance, it was analyzed using a nonparametric method. Since our data were nested as they were collected from 11 sites, a multilevel analysis was used to compare the changes between the two groups. Multilevel analysis, also known as the linear mixed-effects model, allows random intercepts for individuals and sites for differential clustering. Therefore, individuals (participants) and sites were included as a random effect in our multilevel model. In this model, time (three time points including pre-, mid-, post-test) was included as a within-subject (Level 1) parameter, and individuals (Level 2) and sites (Level 3) were included as between-subject parameters. For effect comparison, the group (gardening vs. control) was set as a moderating variable to examine the interaction effect of the time × group. Group-mean centering was adapted to reduce the risk of multicollinearity and increase the ease of interpretation^98^. Effect sizes (Cohen’s d) were calculated for each of the seven psychological distress and well-being measures (MHS:D, MHS:A, CORE, SWLS, MAAS, PSS, and ULS-8). Cohen’s d can be interpreted as small (d = 0.2), medium (d = 0.5), or large (d = 0.8) effect sizes, based on Cohen’s suggestion^99^. Bivariate Pearson correlations between the difference scores (post-score minus pre-score) of each psychological distress and well-being variable and therapeutic alliance were computed to examine the association between changes in each variable and between the therapeutic alliance and changes in psychological distress and well-being variables. According to Cohen’s suggestion, the correlation coefficient indicates a small (r = 0.1), moderate (r = 0.3), or large (r = 0.5) strength of the association between two variables. The *p*-values were adjusted using holm method for multiple correlation analysis to avoid Type I error. After the correlation analysis, a moderation analysis was conducted to examine the impact of the therapeutic alliance on the intervention effect for variables that were significantly correlated with the therapeutic alliance.

Statistical analyses were conducted using R software (version 4.2.2). R software was utilized to conduct multilevel analysis, correlation analysis, and calculate effect size using the “nlme”^100^ and “effsize”^101^ packages. Also, “ggplot2”^102^ package was utilized to visualize the results to facilitate interpretation.

### Supplementary Information

Supplementary Tables.

## Data Availability

The datasets used in the current study are not publicly available due to the ethical standards required by the Institutional Review Board, but are available from the corresponding author on reasonable request.
