# Supplementary material for: The effectiveness of nature-based therapy for community psychological distress and well-being during COVID-19: a multi-site trial
Source: Sci Rep. 2023 Dec 16;13:22370. doi: 10.1038/s41598-023-49702-0 (PMC10724283; doi:10.1038/s41598-023-49702-0)
Supplement: Supplementary file 1 — Supplementary Tables. [file 41598_2023_49702_MOESM1_ESM.docx]

**Table S1.** Effects of study condition (Gardening vs Control) on psychological distress and well-being measures.

|  | **Effects Estimate** | **95% CI** | | | **SE** | **df** | **t** | ***p*-value** |
| --- | --- | --- | --- | --- | --- | --- | --- | --- |
|  |  | **Lower** | | **Upper** |  |  |  |  |
| **MHS:D** |  |  |  | |  |  |  |  |
| Intercept | 11.9849 | 9.5027 | 14.467 | | 1.2627 | 417 | 9.4912 | < 0.0001 |
| Time | -4.0803 | -5.2427 | -2.918 | | 0.5913 | 417 | -6.9005 | < 0.0001 |
| Group | 4.6168 | 2.1523 | 7.0813 | | 1.2512 | 279 | 3.6877 | 0.0003 |
| Time*Group | 2.595 | 1.2014 | 3.9886 | | 0.7090 | 417 | 3.6603 | 0.0003* |
| **MHS:A** |  |  |  | |  |  |  |  |
| Intercept | 12.0457 | 9.6435 | 14.4479 | | 1.2221 | 422 | 9.8564 | < 0.0001 |
| Time | -3.9017 | -5.1945 | -2.6089 | | 0.6577 | 422 | -5.932 | < 0.0001 |
| Group | 5.5126 | 3.6371 | 7.3881 | | 0.9528 | 279 | 5.7859 | < 0.0001 |
| Time*Group | 3.5684 | 2.2108 | 4.9259 | | 0.6906 | 422 | 5.1668 | < 0.0001* |
| **CORE** |  |  |  | |  |  |  |  |
| Intercept | 16.6155 | 15.9136 | 17.3174 | | 0.3571 | 429 | 46.528 | < 0.0001 |
| Time | 1.5679 | 0.9894 | 2.1464 | | 0.2943 | 429 | 5.327 | < 0.0001 |
| Group | -2.4543 | -3.2640 | -1.6446 | | 0.4113 | 279 | -5.967 | < 0.0001 |
| Time*Group | -2.0058 | -2.5768 | -1.4349 | | 0.2905 | 429 | -6.905 | < 0.0001* |
| **SWLS** |  |  |  | |  |  |  |  |
| Intercept | 20.6215 | 19.6195 | 21.6235 | | 0.5098 | 429 | 40.451 | < 0.0001 |
| Time | 2.1897 | 1.0054 | 3.3739 | | 0.6025 | 429 | 3.634 | 0.0003 |
| Group | -3.1973 | -4.9093 | -1.4854 | | 0.8697 | 279 | -3.677 | 0.0003 |
| Time*Group | -2.6656 | -4.0655 | -1.2658 | | 0.7122 | 429 | -3.743 | 0.0002* |
| **MAAS** |  |  |  | |  |  |  |  |
| Intercept | 61.743 | 57.9505 | 65.5358 | | 1.9256 | 248 | 32.064 | < 0.0001 |
| Time | 9.073 | 5.2548 | 12.8919 | | 1.9377 | 222 | 4.683 | < 0.0001 |
| Group | -5.418 | -9.4541 | -1.3811 | | 2.0494 | 248 | -2.644 | 0.0087 |
| Time*Group | -9.207 | -13.984 | -4.4304 | | 2.4239 | 222 | -3.799 | 0.0002* |
| **PSS** |  |  |  | |  |  |  |  |
| Intercept | 17.6697 | 15.8496 | 19.4898 | | 0.9241 | 248 | 19.1209 | < 0.0001 |
| Time | -4.9407 | -6.9617 | -2.9197 | | 1.0255 | 221 | -4.8179 | < 0.0001 |
| Group | 3.9884 | 2.4434 | 5.5335 | | 0.7845 | 248 | 5.0842 | < 0.0001 |
| Time*Group | 5.2994 | 3.2869 | 7.3118 | | 1.0212 | 221 | 5.1896 | < 0.0001* |
| **ULS-8** |  |  |  | |  |  |  |  |
| Intercept | 9.5048 | 8.2866 | 10.7229 | | 0.6184 | 240 | 15.3701 | < 0.0001 |
| Time | -2.9728 | -4.1361 | -1.8094 | | 0.5902 | 216 | -5.0366 | < 0.0001 |
| Group | 1.6126 | 0.1512 | 3.0741 | | 0.7419 | 240 | 2.1736 | 0.0307* |
| Time*Group | 3.566 | 1.7083 | 5.4237 | | 0.9425 | 216 | 3.7835 | 0.0002* |

Note: CI = 95% confidence intervals. SE = Standard Error. df = degree of freedom. t = t-value. **p*-value < 0.01. MHS:D Mental Health Screening Tool for Depressive disorders; MHS:A Mental Health Screening Tool for Anxiety disorders; CORE Core Life Activities Index; SWLS Satisfaction with Life Scale; MAAS Mindful Attention Awareness Scale; PSS Perceived Stress Scale; ULS-8 A short form of the UCLA Loneliness Scale.

**Table S2.** Descriptive statistics and effect sizes for psychological distress and well-being measures.

|  | **Gardening Group** | | | | |  | | **Control Group** | | |  | **Effect**  **Size (Cohen’s *d*)** | **95% CI** | | | **p-value** |
| --- | --- | --- | --- | --- | --- | --- | --- | --- | --- | --- | --- | --- | --- | --- | --- | --- |
|  | **n** | **Pre**  **M(SD)** | **Mid**  **M(SD)** | **Post**  **M(SD)** |  | | **n** | | **Pre**  **M(SD)** | **Post**  **M(SD)** |  |  | **Lower** | **Upper** | |  |
| **MHS:D** | 192 | 17.3 (9.49) | 10.8  (9.38) | 8.43  (9.42) |  | | 99 | | 18.0  (10.7) | 14.2  (10.6) |  | 0.583 | 0.315 | 0.853 | < 0.001 | |
| **MHS:A** | 192 | 16.9  (8.74) | 11.3  (9.01) | 8.49  (9.60) |  | | 99 | | 17.8  (10.1) | 15.7  (10.4) |  | 0.728 | 0.457 | 0.999 | < 0.001 | |
| **CORE** | 192 | 15.0  (3.68) | 16.7  (4.08) | 18.2  (4.39) |  | | 99 | | 14.4  (4.14) | 14.0  (3.63) |  | 1.002 | 0.729 | 1.276 | < 0.001 | |
| **SWLS** | 192 | 18.2  (6.43) | 20.8  (6.78) | 22.8  (7.18) |  | | 99 | | 17.9  (7.02) | 17.3  (6.95) |  | 0.786 | 0.518 | 1.054 | < 0.001 | |
| **MAAS** | 164 | 56.6  (13.2) | - | 66.2  (15.2) |  | | 94 | | 55.6  (14.0) | 56.2  (16.2) |  | 0.645 | 0.364 | 0.925 | < 0.001 | |
| **PSS** | 164 | 20.5  (5.30) | - | 14.9  (6.86) |  | | 93 | | 21.1  (5.52) | 20.7  (5.62) |  | 0.903 | 0.617 | 1.188 | < 0.001 | |
| **ULS-8** | 173 | 11.1  (4.56) | - | 7.93  (4.44) |  | | 78 | | 10.7  (4.31) | 11.0  (4.27) |  | 0.695 | 0.397 | 0.992 | < 0.001 | |

Note: M mean, SD standard deviation, p-values from ANCOVAs entering Marital Status as a covariate. MHS:D Mental Health Screening Tool for Depressive disorders; MHS:A Mental Health Screening Tool for Anxiety disorders; CORE Core Life Activities Index; SWLS Satisfaction with Life Scale; MAAS Mindful Attention Awareness Scale; PSS Perceived Stress Scale; ULS-8 A short form of the UCLA Loneliness Scale.

**Table S3**. The example of therapeutic gardening program.

| **Session** | **Activity** | **Session** | **Activity** |
| --- | --- | --- | --- |
| 1 | Introduction | 16 | Potting 2: Setting up mini garden |
| 2 | Preparing gardening | 17 | Event 3: Making potpourri |
| 3 | Setting up garden 1: making bed | 18 | Designing garden 1: Drawing the garden |
| 4 | Setting up garden 2: Blending soil | 19 | Designing garden 2: Planting shrub |
| 5 | Setting up garden 3: Fertilizing | 20 | Designing garden 3: Planting herbs and bulbs |
| 6 | Setting up garden 4: Making compost | 21 | Designing garden 4: Planting vines |
| 7 | Plant Propagation 1: Seed propagation | 22 | Designing garden 5: Mulching |
| 8 | Plant Propagation 2: Asexual reproduction | 23 | Reporting 1: Monitoring the garden |
| 9 | Planting 1: Tree planting | 24 | Reporting 2: Making plant labels |
| 10 | Planting 2: Potting plants | 25 | Pruning plants |
| 11 | Planting 3: Transplanting | 26 | Getting rid of weeds |
| 12 | Event 1: Enjoying herb tea | 27 | Pest control |
| 13 | Lecture: Animals, birds, insects in garden | 28 | Event 4: Yoga and picnic in the garden |
| 14 | Event 2: Flower arrangement | 29 | Preparing garden for winter |
| 15 | Potting 1: Repotting | 30 | Event 5: Garden party |
